# Combinatorial Discriminant Analysis Applied to RNAseq Data Reveals a Set of 10 Transcripts as Signatures of Exposure of Cattle to *Mycobacterium avium subsp. paratuberculosis*

**DOI:** 10.3390/ani10020253

**Published:** 2020-02-05

**Authors:** Michela Malvisi, Nico Curti, Daniel Remondini, Maria Grazia De Iorio, Fiorentina Palazzo, Gustavo Gandini, Silvia Vitali, Michele Polli, John L. Williams, Giulietta Minozzi

**Affiliations:** 1 Parco Tecnologico Padano, 26900 Lodi, Italy; michela.malvisi@yahoo.com; 2 Department of Veterinary Medicine DIMEVET, University of Milan, 20133 Milan, Italy; mariagrazia.deiorio1993@gmail.com (M.G.D.I.); Gustavo.gandini@unimi.it (G.G.); Michele.polli@unimi.it (M.P.); 3 Department of Physics and Astronomy, University di Bologna, 40126 Bologna, Italy; nico.curti2@unibo.it (N.C.); silvia.vitali4@unibo.it (S.V.); 4 Faculty of Bioscience and Technology for Food, Agriculture and Environment, University of Teramo, 64100 Teramo, Italy; fpalazzo@unite.it; 5 Davies Research Centre, School of Animal and Veterinary Sciences, University of Adelaide, Roseworthy, South Australia 5005, Australia; john.williams01@adelaide.edu.au

**Keywords:** Johne’s disease, bovine, *Mycobacterium avium subsp. paratuberculosis*, RNAseq, combinatorial discriminant analysis, biomarker discovery

## Abstract

**Simple Summary:**

Paratuberculosis or Johne’s disease (JD) in cattle is a chronic granulomatous gastroenteritis caused by infection with *Mycobacterium avium subspecies paratuberculosis* (MAP). JD is not treatable; therefore, the early identification of infected animals is a key point in reducing its incidence worldwide. In this paper, combinatorial discriminant analysis was applied to transcriptomic data to find a small set of differentially expressed genes able to discriminate between exposed cattle from non-exposed animals. Results of the discriminant analysis identified 10 transcripts that differentiate between ELISA-negative animals belonging to paratuberculosis-positive herds that were potentially exposed to MAP and negative-unexposed animals belonging to paratuberculosis-negative herds. The 10 transcript signature was also used for the classification of 5 cattle positive-for-MAP infection samples, with the underlying hypothesis that their transcriptional profile should be closer to potentially exposed animals than to controls. The same set of 10 transcripts was able to differentiate ELISA-negative unexposed animals from positive animals based on the results of the ELISA test for bovine paratuberculosis and faecal culture. In conclusion, these findings suggest the possible use of the identified RNA expression signature as a new diagnostic test for paratuberculosis.

**Abstract:**

Paratuberculosis or Johne’s disease in cattle is a chronic granulomatous gastroenteritis caused by infection with *Mycobacterium avium subspecies paratuberculosis* (MAP). Paratuberculosis is not treatable; therefore, the early identification and isolation of infected animals is a key point to reduce its incidence. In this paper, we analyse RNAseq experimental data of 5 ELISA-negative cattle exposed to MAP in a positive herd, compared to 5 negative-unexposed controls. The purpose was to find a small set of differentially expressed genes able to discriminate between exposed animals in a preclinical phase from non-exposed controls. Our results identified 10 transcripts that differentiate between ELISA-negative, clinically healthy, and exposed animals belonging to paratuberculosis-positive herds and negative-unexposed animals. Of the 10 transcripts, five (*TRPV4*, *RIC8B*, *IL5RA*, *ERF*, *CDC40*) showed significant differential expression between the three groups while the remaining 5 (*RDM1*, *EPHX1*, *STAU1*, *TLE1*, *ASB8*) did not show a significant difference in at least one of the pairwise comparisons. When tested in a larger cohort, these findings may contribute to the development of a new diagnostic test for paratuberculosis based on a gene expression signature. Such a diagnostic tool could allow early interventions to reduce the risk of the infection spreading.

## 1. Introduction

Paratuberculosis or Johne’s disease (JD) in cattle is a chronic granulomatous gastroenteritis caused by infection with *Mycobacterium avium subspecies paratuberculosis* (MAP) [1,2]. JD is present worldwide, is a welfare issue and causes significant economic losses. Cattle are usually infected as young calves but typically do not show clinical signs before 24 months of age; however, not all infected animals progress to clinical disease [1]. During this extended period of silent infection, animals may shed MAP in their feces into the environment before infection can be diagnosed with the current methods. JD is not treatable; therefore, the early identification and isolation of infected animals before they start shedding the bacteria is a key point to reduce its incidence in cattle herds worldwide. 

An association between MAP and Crohn’s disease (CD) in humans has been suggested. Both JD and CD share similar symptoms and lesions, and MAP has been identified in tissue isolates of CD patients; however, the causative association is still controversial [3,4]. Given the economic losses and welfare concerns for livestock, and possible human health risk, the research interest in JD has been focused on the substantial difficulty in early diagnosis of infected animals and the exploration of potential new diagnostic techniques [5].

Several studies have analyzed transcriptomic profiles of both RNA and MicroRNA in cattle to identify indicators of MAP infection status [6,7,8,9,10]. One of these studies [9] used RNAseq transcriptomic profiling of bovine monocyte-derived macrophages (MDM) cultured in vitro and infected with the L1 MAP strain and identified 3212 differentially expressed transcripts at 2, 6 and 24 h post-infection. Understanding the host–pathogen interaction in the initial phases is essential to understand the biology of infection. Nevertheless, the transcriptomic profile of the later phases of the disease may guide the identification of changes occurring in the pre-clinical and clinical phase of disease. 

In a previous study conducted by the authors of the current manuscript, the whole blood transcriptomic profiles of 5 animals serologically positive to the ELISA test for MAP (PP), 5 ELISA-negative potentially exposed animals (NP) and 5 serologically negative unexposed control animals (NN) were described [8]. In total, in our previous work, we identified 258 differentially expressed genes (DE) between the three groups: 162 were differentially expressed between PP vs. NN, 94 between NP vs. NN and 2 between PP and NP. 

In the context of high-throughput data analysis, a challenge is defining an optimal choice of variables (a “signature”) to classify groups of samples or regress trends with optimal performance and minimum dimensionality. Usually, high-throughput omics data (e.g., transcriptomics, genomics, methylomics) provide datasets with tens to hundreds of samples, and often 1000 times larger numbers of variables [11]. The objective of dimensionality reduction through the choice of an optimal signature is twofold: (1) the identification of relevant variables that should separate the signal from the noise (i.e., variables not significantly associated to, or descriptive of the studied process); and (2) in a practical context, it is important to establish future diagnostic criteria that can be implemented in cheap and simple toolkits, such as PCR cards or dedicated microarray chips, that usually test a small number of transcripts (ranging from tens to hundreds, at most). We have recently applied dimensionality reduction methods in other high-throughput gene expression contexts, with, e.g., 10^4^ transcripts, and obtained an approximately 1000-fold reduction of the number of transcripts required while retaining the discriminatory signatures [12,13,14]. These studies show the feasibility of such approaches, which could be translated into diagnostics for clinical practice, or to suggest different therapeutic strategies based on the patient’s genomic profile (in the scope of so-called personalized medicine).

The purpose of the present study was to apply machine learning approaches, specifically a custom algorithm involving Combinatorial Discriminant Analysis, to RNAseq data on cattle belonging to three groups based on different MAP infection status [8], to find an optimal low-dimensional signature able to discriminate between ELISA-negative potentially exposed animals compared to negative-unexposed controls. Such a signature, when validated in a larger cohort of animals, could aid early interventions to reduce sanitary and economic burdens and to reduce the risk of JD infection spreading. 

## 2. Materials and Methods

### 2.1. Animal Resource

Holstein dairy cows (15 animals in total) were selected from two herds, one MAP-free (verified by ELISA and the absence of clinical cases) and the other positive for MAP (verified by ELISA, faecal culture and the presence of clinical cases). From the positive herd, 5 positive (PP) cows based on ELISA test results and 5 ELISA-negative, potentially exposed (NP) animals were chosen. All animals were 4 to 5 years old, had a body condition score (BCS) of 3 points and were at 170 to 190 days in milk (DIM) when sampled. The five ELISA positive subjects (PP) were confirmed by faecal culture. Five negative non-exposed control animals (NN) were selected from the negative herd, matched for age, stage of lactation, BCS and DIM with the positive and exposed animals [8]. 

### 2.2. Sample Preparation, RNA Extraction and Quality Control

Samples preparation, DNA extraction, library preparation and quality control are detailed in Malvisi et al. (2016) [8]. In brief, samples used were taken during obligatory routine animal sanitary controls by an authorised veterinarian. No ethical approval was required, in compliance with the European Directive 2010/63/UE and the Italian Regulation D. Lgs n. 26/2014. Whole blood was collected into PAXgene^®^ Blood RNA tubes (PreAnalytiX GmbH). Total RNA was extracted using the PAXgene^®^ Blood miRNA kit (PreAnalytiX GmbH). RNA concentration was measured by a NanoDrop^™^ 1000 spectrophotometer (Thermo Scientific) and RNA integrity was assessed with an Agilent 2200 TapeStation system (Agilent Technologies).

### 2.3. RNA-Seq Library Preparation and Sequencing

Libraries were prepared with the Illumina Truseq RNA sample prep kit (Illumina Inc., USA), size and yield were evaluated using an Agilent TapeStation 2200. Fifteen libraries were prepared and equimolar amounts of 5 samples were mixed before NaOH denaturation. Each of the pools was run in a lane of a Hiseq Flowcell. The Illumina Truseq PE cluster kit v3 was used to generate clusters on the grafted Illumina Flowcell and the hybridised libraries were sequenced on a Hiseq2000 with a 100 cycles of paired-end sequencing module using the Truseq SBS kit v3 (Illumina Inc., San Diego, CA, USA).

### 2.4. Availability of Data

All data generated or analysed during this study are available online (GEO reference number GSE130124). Furthermore, all data are available on request. In addition, all transcript counts per sample are given as supplementary information files GSE130124.

### 2.5. RNA-Seq Data Analysis

Preliminary quality control of raw reads was carried out with FastQC software v0.11.2 [15]. Illumina raw sequences were trimmed using Trimmomatic [16] and PCR primers and Illumina adapter sequences were removed. Minimum base quality 15 over a 4-base sliding window was required, then only sequences longer than 36 nucleotides were included in the downstream analysis. Reads which successfully passed trimming were mapped against the *Bos taurus* UMD3.1.68 reference genome sequence, using STAR [17] aligner to obtain BAM alignment files. The BAM files were sorted and indexed using Samtools [18]. In order to quantify counts for each sample, a list of genes and relative abundance of mapping reads were extracted using htseq-count [19]. These count files were used for downstream statistical analysis. Data from Malvisi et al. [8] was used (GEO reference number GSE 130124).

### 2.6. Signature Identification

A combinatorial discriminant analysis was conducted on the RNAseq data of the 15 samples derived from the study published by the authors [8], which has been previously analysed using a traditional pairwise comparison between groups at a single gene level. In detail, the dataset comprised 15,036 transcripts from 15 samples, classified as “serologically negative non-exposed cows/healthy” (5 samples, labelled as NN), “serologically negative exposed cows” (5 samples, NP) and “serologically positive/clinical cows” (5 samples, PP). Only transcripts with non-zero counts for all samples were considered, reducing the dataset to 13,529 transcripts.

A custom feature selection algorithm based on Quadratic Discriminant Analysis was used to identify an optimal low-dimensional signature able to discriminate between NN and NP samples based on a method developed by the authors, described in [20,21,22], which had been used previously to analyse biological and social data [13,14,21]. The algorithm evaluates all possible pairs of transcripts which it groups onto a transcript–transcript network weighted by classification performance. The putative signatures are obtained through thresholding based on classification performance for the disconnected components resulting from the initial weighted network. However, optimal performance of the algorithm requires a larger sample size than that available in this study [14,20,21,22], to allow more performance threshold values to be tested. The smaller sample size used here tends to generate a single giant component with a large number of transcripts. Thus, to reduce the dimensionality of the classification signature, (1) the most central nodes of the giant component were considered, with a combination of two centrality measures (betweenness centrality [23] and clustering coefficient [24]) that reduced the signature size to 123 transcripts; (2) the pendant nodes (i.e., with only one link) were recursively removed, until no further removals were allowed, leading to a final signature of 10 transcripts. In order to validate the classification performance of the signature, as the low number of samples did not allow the application of procedures such as robust k-fold cross-validation with a subset of samples of the same classes, we used the signature to classify the PP data, which was not used for signature identification, with the hypothesis that these samples should be classified more closely to the NP samples than the NN ELISA-negative samples. 

## 3. Results

### 3.1. Results of the NGS Pipeline Analysis

The results of the RNAseq analysis pipeline and list of differentially expressed genes between the three animal groups (PP, NP and NN) are described by Malvisi et al. [8]. In summary, RNAseq data for whole blood of the 5 PP, 5 NP and 5 NN animals identified 12,366 genes, of which 258 were differentially expressed (DE) in the three comparisons: 162 genes were DE comparing PP vs. NN, 94 genes for NP vs. NN and 2 genes for PP vs. NP. Differential expression was defined as log_2_ fold change value above 1 or below -1 and a FDR threshold of 0.05. The complete list of DE genes for each comparison, derived from Malvisi et al. [8], is given in Supplementary tables (Tables S1–S3).

### 3.2. Results of the Signature Analysis 

Starting from the top-performing pairs of transcripts, with the first thresholding step of the weighted transcript network (see the Methods section) we obtained an initial signature of 123 transcripts capable of correctly classifying 4 out of the 5 NN samples (80%) and all 5 NP samples (100% performance). The average performance was therefore 90%, with a Matthews correlation coefficient MCC = 0.82 (a complete list of the 123 transcripts is given in Supplementary Table S4). 

**Figure 1:**
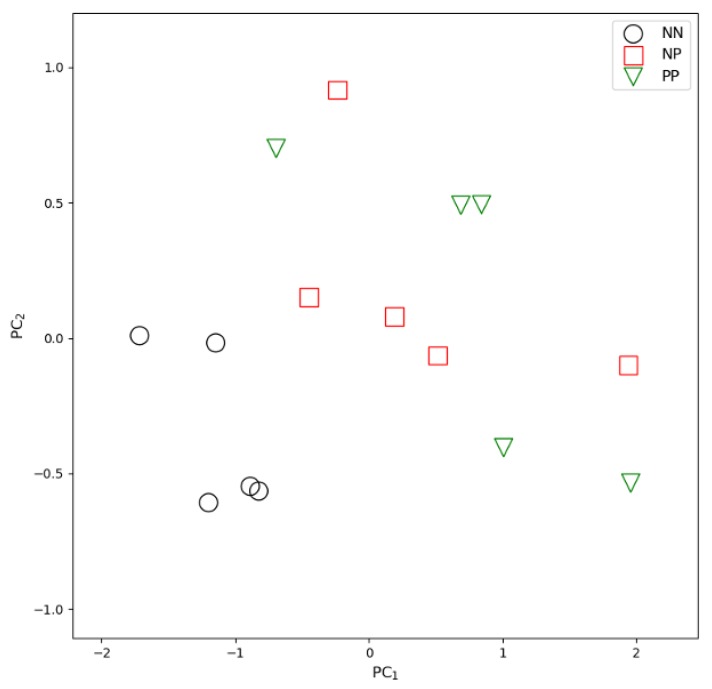
Principal Component Analysis (first 2 components) applied to the three groups of animals analyzed by RNAseq analysis (5 animals serologically positive to the ELISA test for MAP (PP), 5 ELISA-negative potentially exposed animals (NP) and 5 serologically negative unexposed control animals (NN), based on the RNA-seq profile of the 10 signature transcripts.

Processing the 123-transcript network by removing all pendant nodes, we obtained a final signature with 10 transcripts (Table 1) with a 100% performance classifying all NN and NP samples (Figure 1). Five transcripts of the signature show significant difference in the pairwise comparisons between the NN and NP groups (TRPV4, RIC8B, IL5RA, ERF and CDC40, see Table 1). The minimum log_2_ fold change difference was −0.016 (*ASB8* gene transcript) while maximum was −1.556 for the *IL5RA* transcript.

**Figure 2:**
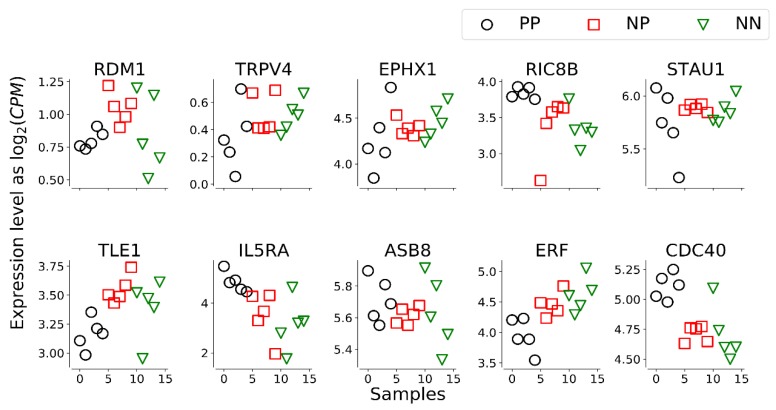
Plot of transcript levels for the 10 genes belonging to the signature. Some transcripts (EPHX1, IL5RA, ERF, CDC40) show a clear trend when moving from 5 animals serologically positive to the ELISA test for MAP (PP), to 5 ELISA-negative potentially exposed animals (NP) and 5 serologically negative unexposed control animals (NN).

**Figure 3:**
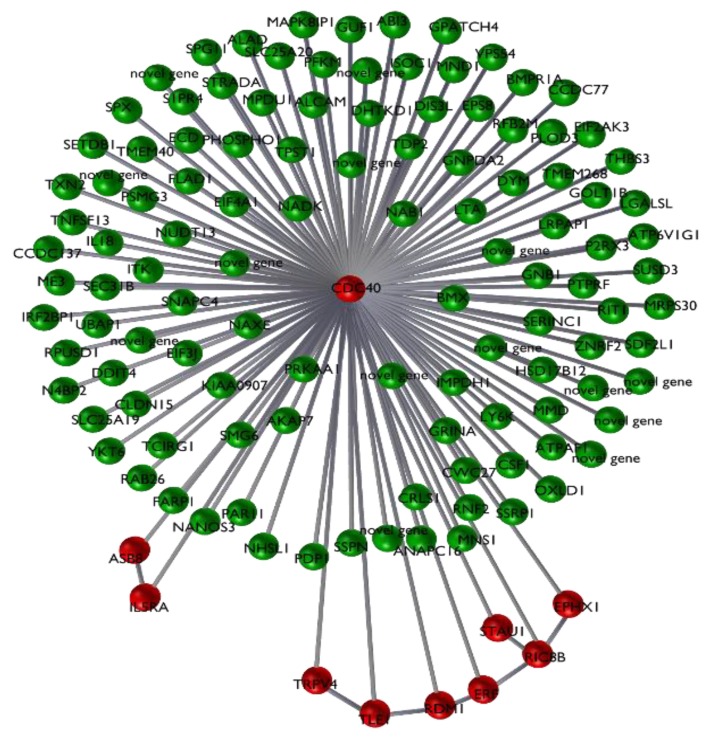
Plot of the 123-transcript network (green nodes), with a detail of the 10-probe signature (red nodes).

The first two principal components in a PCA of the 10-transcript signature (Figure 1) highlight a clear separation between NN and NP + PP samples. Moreover, the expression levels of the single transcripts of the signature show a progressive increase or decrease from a healthy (NN) samples to a positive (PP) samples, passing through the exposed (NP) sample class, specifically for EPHX1, IL5RA, ERF and CDC40 (Figure 2). In the 123-transcript network, all nodes are directly connected to a central 10-transcript core, while only these core 10 transcripts, which form the signature, are connected to each other (see Figure 3).

To further validate the goodness of the signature, we generated 10,000 different signatures with 10 randomly chosen transcripts, and then applied a Leave-One-Out cross validation procedure to quantify the classification performance of these signatures. Only 50 of these signatures (i.e., the 0.5% of the total random signatures) produced a better classification performance than that achieved using the core 10 transcripts. Thus, it is unlikely to obtain a signature of similar performance with the same number of transcripts by pure chance. The statistical significance of the signature identified using our feature selection algorithm would be improved by extending the data set.

## 4. Discussion

Earlier detection of subclinical disease is urgently required to enhance control of MAP. However, the mechanisms involved in progressive evolution of JD phases are still unknown, which limits the development, identification and sensitivity of diagnostic tools. Gene expression profiling from peripheral blood has been shown to discriminate patients affected by Crohn’s Disease, ulcerative colitis and non-inflammatory diarrheal disorders [25]. Recently, a smaller panel of only 6 genes that are differentially expressed during inflammatory bowel disease, identified by supervised learning methods, were shown to discriminate between the different stages of the disease such as mild severity, moderate-to-severe, inactive forms of Crohn’ s disease and ulcerative colitis with very high sensitivity [26]. 

Further examples involve the development of a small panel, composed of seven differentially expressed genes (*ANXA3*, *CLEC4D*, *LMNB1*, *PRRG4*, *TNFAIP6*, *VNN1* and *IL2RB*), able to discriminant between subjects according to their relative risk for colorectal cancer in an average-risk population based on 642 samples [27]. Therefore, mRNA expression profiling is seen as a potential source of biomarkers for early diagnosis of JD [7,28,29]. Several DE transcripts have been identified in whole blood of Holstein–Friesian calves challenged with a high or low dose of MAP three months after inoculation and control calves [28]. Furthermore, the same authors identified that calves receiving a high dose of MAP showed more up-regulated transcripts. In a study of a mixed population of Holstein and Holstein Red calves aged between 2 to 4 months inoculated experimentally with MAP, 63 DE genes were identified early in infection [29]. Of the genes identified in these studies, some have a role in immune function (*IL7*, *TRIM13*, *ICOS*, *CD46*), but none are in common with the 10 genes forming the core signature identified in the present study. However, experimental designs differed, and responses of young calves exposed to experimental MAP infection may not coincide with those of naturally infected (PP) adult cattle examined here. 

The aims of high-throughput discovery studies are to gain deeper biological insight into the underlying phenomena (as in Malvisi et al. [8]), or to find an optimal subset of variables able to describe the phenomenon (e.g., in terms of sample classification or stratification) by means of robust statistical analyses. In this paper, we applied a custom dimensionality reduction method to the initial set of variables to obtain a multivariate signature, with optimal performance in terms of sample classification using a small subset of variables. The authors have successfully applied this approach in other contexts involving high-throughput transcriptomics data. These analyses allowed samples to be stratified and different therapeutic strategies based on individual genomic profiling to be proposed, and in some cases, specific pathways associated to the clinical outcome to be identified [12,13,14]. The methodology identifies DE genes that individually may not be significant or biologically relevant, but that taken together as a group, gain discriminative value.

Results of supervised classification analysis of the RNAseq data reported here identified a signature of 10 transcripts that is able to differentiate between ELISA-negative, likely exposed, but clinically healthy animals belonging to JD positive herds (NP), and negative-unexposed animals (NN). Furthermore, the same set of 10 transcripts differentiate negative-unexposed (NN) animals from positive animals defined by the ELISA test for bovine paratuberculosis and faecal culture (PP). 

Within the 10 transcripts of the signature, 5 genes (*TRPV4*, *RIC8B*, *IL5RA*, *ERF* and *CDC40*) showed significant differential expression between the NN, NP and/or PP groups, while the remaining 5 transcripts (*RDM1*, *EPHX1*, *STAU1*, *TLE1*, *ASB8*) did not show a significant difference in least in one of the pairwise comparisons (Supplementary Table S4).

Among the significantly differentially expressed genes in the signature, the cell division cycle 40 gene (*CDC40*) showed the smallest fold change between classes, this gene is the most central node of the signature network associated with JD health status (Figure 3). *CDC40* is located on BTA9, and was under-expressed in the NP and PP groups, compared with the NN group. *CDC40* has been shown to be involved in clathrin-mediated endocytosis [30]. Clathrin is the best characterised coat protein involved in the endocytosis process, specifically in receptor-mediated internalization [31]. *Mycobacterium paratuberculosis* enters the host macrophages, its primary target cell, and manages to survive within the phagosome [32]. It is possible that the under-expression of *CDC40* in infected and sick animals, compared to unexposed animals, may facilitate the survival of the pathogen within the host target cell. In addition to the *CDC40* gene, other transcripts in the core set are involved with immune response mechanisms; these include *IL5RA*, *ERF* and *TRPV4*. These genes potentially have functions related to the biology of the progression of JD. Interleukin 5 receptor subunit alpha (*IL5RA*) is under-expressed in infected NP and serologically positive PP animals compared with unexposed NN animals. IL5 is a cytokine that acts as a growth and differentiation factor for both B cells and eosinophils. Recently *IL5RA* was found to be under-expressed in human tuberculosis (TB) patients compared with healthy controls [33]. Further, after 2 and 6 months of treatment for TB, *IL5RA* was found overexpressed in the treated patient group compared to the healthy controls, indicating a reactivation of the immune cells [33]. This is in line with the present study, where this gene is significantly under-expressed in the para-tuberculosis infected and exposed samples compared with controls. *IL5RA* may possibly be incorporated into a diagnostic tool to monitor disease progression. It is worth noting that *IL5RA* belongs to the most affected gene network associated with PP vs. NN [8]. This network is enriched in genes associated with the inflammatory response. *IL5RA* shows several connections with other DE genes in the study, indicating that *IL5RA* acts as a central node [8]. The ETS2-repressor factor gene (*ERF*) encodes a transcriptional repressor factor that is ubiquitously expressed and known to have tumor suppressor activity [32]. *ERF* is one of the five genes that was over-expressed in the PP and NP group compared with the NN group (with log_2_ fold change of 0.68 and 0.55 log_2_ fold change, respectively, between the PP and NP groups vs. the NN group). However, *ERF* was not differentially expressed between the PP and NP groups (0.126 log_2_ fold change and 0.48 p-value), although it was one of the five genes out of 10 that showed statistical significance in the pairwise comparisons [8]. Several studies suggest a role of *ERF* in the immune response and macrophage biology and is a candidate for ParaTB diagnostics [34]. It has been shown that the human Erf protein decreases the expression of mouse c-Myc mRNA in RAS-3T3 cells [34]. In normal conditions, c-Myc is a transcription factor that binds to more than 10% of human promoters and regulates several crucial cell functions [34]. ERF binding to the c-Myc promoter has been shown to reduce *c-Myc* transcription [34], and overexpression of *ERF* causes down-regulation of *c-Myc*. Interestingly, *c-Myc* is a key factor in macrophage differentiation [35]. Inhibition of *c-Myc* expression may prevent macrophage activation induced by infecting bacteria. Furthermore, the overexpression of *ERF* and repression of *c-Myc* is consistent with the under-expression we observed of *IL-15RA* [8]. The protein encoded by the *c-Myc* has been shown to affect IL-15RA mRNA levels in murine studies [36]. The Transient Receptor Potential Vanilloid 4 gene (*TRPV4* located on chromosome 17 in cattle), was overexpressed in the NP and PP animals compared with the unexposed animals. *TRPV4* encodes a non-selective cation channel (for, e.g., sodium, calcium, potassium) that is expressed widely and is involved in numerous metabolic processes in humans, including inflammation, where it contributes to disease progression [37]. Interestingly, TRPV4 has a role in gastrointestinal inflammatory diseases such as ulcerative colitis (UC) and Crohn’s disease (CD), which are part of a group of inflammatory bowel diseases (IBD) [38]. *TRPV4* is expressed widely in the intestinal tract and activation of *TRPV4* causes the release of pro-inflammatory cytokines and an inflammatory response a few hours after administration [38]. Furthermore, over-expression of *TRPV4* has been observed in murine inflamed colon tissues compared with healthy controls [38]. This suggests that as the inflammatory environment increases, both *TRPV4* expression and the activation of the non-selective cation channel help to maintain the inflammatory state. This is consistent with the increased level of expression of *TRPV4* in NP and PP animals compared to the unexposed animals (NN) seen in the present study, and suggests that *TRPV4* should be included in the set of diagnostic markers for paratuberculosis.

The other transcripts identified by the discriminant analysis do not show an obvious role in JD or bacterial infections in general. The *RDM1* (RAD52 motif containing 1) gene codes for a protein that is involved in a DNA damage repair pathway, and in the cellular response to cisplatin, a drug commonly used in chemotherapy [39,40]. STAU1 is a member of the family of double-stranded RNA (dsRNA)-binding proteins involved in the transport of mRNAs to different organelles or cell compartments. In cattle, the protein has been shown to have a role during mammalian oocyte maturation and early embryogenesis [41].

*EPHX1* (Epoxide Hydrolase 1) codes for an enzyme involved in the degradation of aromatic compounds to less reactive and more water-soluble compounds [42]. Mutations of *EPHX1* may alter its enzymatic function and lead to Familiar Hypercholanemia, a genetic disorder characterized by high serum bile acid concentrations, itching, and fat malabsorption [43]. *ASB8* (Ankyrin Repeat and SOCS Box Containing 8) has a putative role in the growth and proliferation of lung cancer, possibly as a positive regulator [44]. The *TLE1* (TLE Family Member 1, Transcriptional Corepressor) gene product is involved in hemopoiesis, epithelial and neuronal differentiation and has a role in repressing cell differentiation [45]. TLE1 has been used as a immunohistochemical marker for Synovial Sarcoma, an adult soft tissue sarcoma [46]. Lastly, the *RIC8B* (RIC8 Guanine Nucleotide Exchange Factor B) gene product is known to interact with G-alpha proteins [47] and potentiates odorant receptor signal transduction by increasing G (olf)- alpha-dependent cAMP accumulation [48].

In summary, the results of the discriminant analysis identified a 10-gene signature, the expression levels of which could discriminate between the exposed and sero-converted animals. Not all of the 10 genes were DE or relate to immune function. To further validate these findings, a larger group of animals should be tested. The study was limited by the unknown exposure status of the NP group, which gives uncertainty in the interpretation of the signature identified, and by the lack of a longitudinal information on the disease outcome of the NP group. 

## 5. Conclusions

In conclusion, the discriminant analysis described here identified a 10-gene signature, the expression levels of which could discriminate between exposed and sero-converted animals and suggest the possible use of RNA expression analysis as a new diagnostic test for JD. In particular, the approach may be able to identify infected or/exposed animals prior to sero-conversion and a positive ELISA test result. However, further tests for specificity and validation in a larger cohort are required.

## Figures and Tables

**Table 1:** The set of 10 transcripts that best discriminate between 5 animals serologically positive to the ELISA test for MAP (PP), 5 ELISA-negative potentially exposed (NP) and 5 serologically negative unexposed control animals (NN), and their corresponding pairwise log_2_ fold change in the PP vs. NN, NP vs. NN and PP vs. NP comparisons.

Gent Name	PP vs. NN	NP vs. NN	PP vs. NP
*RDM1*	0.153	0.492	−0.338
*TRPV4*	1.535 *	1.457 *	0.948 *
*EPHX1*	0.132	0.104	0.028
*RIC8B*	−0.538 *	−0.415	−0.122
*STAU1*	0.081	0.155	−0.074
*TLE1*	0.238	0.445	−0.207
*IL5RA*	−1.556 *	−1.191	−0.366
*ASB8*	−0.095	−0.078	−0.016
*ERF*	0.681 *	0.554	0.126
*CDC40*	−0.429 *	−0.387	−0.042

* Log_2_ fold changes that show significant difference (*p* < 0.05) in the comparison tests (Student’s *t*-test).

## Supplementary Materials

The following are available online at https://www.mdpi.com/2076-2615/10/2/253/s1, Table S1. Differentially expressed genes in the positive subject when compared with the control group. Genes with an FDR < 0.05 and a Log2 fold change (LogFC) <−1 or >1 were considered as differentially expressed. Table S2. Differentially expressed genes in the exposed subject when compared with the control group. Genes with an FDR < 0.05 and a Log2 fold change (Log2FC) <−1 or >1 were considered as differentially expressed. Table S3. Differentially expressed genes in the positive subject when compared with the exposed group. Genes with an FDR < 0.05 and a Log2 fold change (Log2FC) <−1 or >1 were considered as differentially expressed. Table S4. List of 123 transcripts identified by Combinatorial Discriminant Analysis (CDA) that discriminates between cattle infected and non-infected by *Mycobacterium avium* ssp. *Paratuberculosis*.
