# Supplementary material for: Combinatorial Discriminant Analysis Applied to RNAseq Data Reveals a Set of 10 Transcripts as Signatures of Exposure of Cattle to Mycobacterium avium subsp. paratuberculosis
Source: Animals (Basel). 2020 Feb 5;10(2):253. doi: 10.3390/ani10020253 (PMC7070263; doi:10.3390/ani10020253)
Supplement: Supplementary file 1 [file animals-10-00253-s001.pdf]

Table S1. Differentially expressed genes in the positive subject when compared with the control group. Genes with an FDR<0,05 and a Log2 fold change (LogFC) <-1 or >1 were considered as differentially expressed.

Sequence ID	Gene symbol	LogFC	FDR
ENSBTAG00000002942	SLC2A10	3.60	0.012620
ENSBTAG00000039283	novel gene	3.38	0.000744
ENSBTAG00000031849	TMEM119	2.07	0.003607
ENSBTAG00000033897	LOC784762	1.97	0.012135
ENSBTAG00000022275	novel gene	1.96	0.000044
ENSBTAG00000039059	GP1BB	1.88	0.020010
ENSBTAG00000047836	novel gene	1.87	0.000005
ENSBTAG00000038913	novel gene	1.83	0.001073
ENSBTAG00000004526	TPT1	1.82	0.000021
ENSBTAG00000011299	novel gene	1.75	0.000000
ENSBTAG00000031453	CCM2L	1.73	0.018149
ENSBTAG00000045673	LOC613345	1.69	0.000468
ENSBTAG00000003086	RPLP2	1.63	0.001140
ENSBTAG00000031795	novel gene	1.62	0.007453
ENSBTAG00000014824	MMP14	1.61	0.019666
ENSBTAG00000040210	novel gene	1.59	0.000108
ENSBTAG00000032702	novel gene	1.57	0.000005
ENSBTAG00000046010	BHLHA15	1.53	0.003726
ENSBTAG00000029982	MIR142	1.52	0.001973
ENSBTAG00000009737	LTC4S	1.50	0.043235
ENSBTAG00000047700	novel gene	1.50	0.007155
ENSBTAG00000015040	LOC100141215	1.49	0.008841
ENSBTAG00000046245	novel gene	1.48	0.000005
ENSBTAG00000012219	CSPG4	1.42	0.022408
ENSBTAG00000003152	novel gene	1.41	0.019666
ENSBTAG00000047508	ZNF580	1.36	0.000114
ENSBTAG00000010060	SLC43A3	1.35	0.027852
ENSBTAG00000014666	GP9	1.35	0.040135
ENSBTAG00000017113	ANKRD33	1.33	0.014076
ENSBTAG00000046100	novel gene	1.32	0.000006
ENSBTAG00000026489	TNFRSF13C	1.30	0.000052
ENSBTAG00000017981	ADRB3	1.29	0.014180
ENSBTAG00000029996	MIR27A	1.29	0.033276
ENSBTAG00000024280	novel gene	1.29	0.009768
ENSBTAG00000014358	EVA1B	1.29	0.000799
ENSBTAG00000021338	OAF	1.28	0.003116
ENSBTAG00000008542	SSPO	1.27	0.020815
ENSBTAG00000015405	DCHS1	1.27	0.009681
ENSBTAG00000046803	GAS1	1.27	0.000744
ENSBTAG00000007693	SLC45A3	1.23	0.037975
ENSBTAG00000019095	CCR10	1.23	0.012081

ENSBTAG00000024814	B4GALNT4	1.23	0.023114
ENSBTAG00000026501	CYP2D14	1.22	0.000008
ENSBTAG00000003923	GPBAR1	1.21	0.000049
ENSBTAG00000017405	RORC	1.21	0.003315
ENSBTAG00000047926	OVCA2	1.21	0.033083
ENSBTAG00000023365	LOC100297056	1.20	0.042990
ENSBTAG00000043971	NOTCH3	1.20	0.013604
ENSBTAG00000018016	NUPR1	1.19	0.038395
ENSBTAG00000027386	novel gene	1.19	0.044830
ENSBTAG00000020341	MOV10L1	1.18	0.028551
ENSBTAG00000027654	EIF4EBP1	1.18	0.000009
ENSBTAG00000011680	PAPLN	1.18	0.005871
ENSBTAG00000018870	OTOA	1.18	0.043235
ENSBTAG00000018920	TRIM47	1.18	0.000154
ENSBTAG00000025130	HIC1	1.17	0.034922
ENSBTAG00000038055	KLF16	1.17	0.000021
ENSBTAG00000019844	ZNF467	1.16	0.000082
ENSBTAG00000014705	HES4	1.16	0.010625
ENSBTAG00000019249	HS3ST6	1.16	0.020815
ENSBTAG00000047659	novel gene	1.15	0.005161
ENSBTAG00000021602	CTTNBP2NL	1.12	0.018130
ENSBTAG00000047538	RIMBP3B	1.11	0.042399
ENSBTAG00000046140	NPDC1	1.10	0.005763
ENSBTAG00000015108	USHBP1	1.10	0.014421
ENSBTAG00000044158	LDLRAD3	1.10	0.043561
ENSBTAG00000005934	TTYH3	1.10	0.000160
ENSBTAG00000037757	EBF4	1.09	0.017789
ENSBTAG00000001824	SLC2A6	1.09	0.000005
ENSBTAG00000001814	PLXND1	1.08	0.002506
ENSBTAG00000030521	IER5L	1.06	0.000005
ENSBTAG00000046486	novel gene	1.06	0.001277
ENSBTAG00000007253	TSPAN33	1.06	0.003840
ENSBTAG00000003607	FAM20C	1.05	0.008322
ENSBTAG00000014983	IL23R	1.05	0.043014
ENSBTAG00000020532	novel gene	1.04	0.040725
ENSBTAG00000000042	PYCR1	1.04	0.011853
ENSBTAG00000007375	MIF	1.03	0.000055
ENSBTAG00000046111	ZNF444	1.02	0.000189
ENSBTAG00000000244	NRXN2	1.02	0.005763
ENSBTAG00000004093	TUBB2B	1.02	0.000618
ENSBTAG00000021755	novel gene	1.01	0.000723
ENSBTAG00000021013	TUBB4A	1.01	0.047503
ENSBTAG00000034613	MROH6	1.00	0.012189
ENSBTAG00000016525	ITGA1	-1.00	0.024274
ENSBTAG00000001457	C20H5orf28	-1.00	0.012135
ENSBTAG00000046323	novel gene	-1.02	0.035471

ENSBTAG00000031388	DYX1C1	-1.02	0.040227
ENSBTAG00000040051	novel gene	-1.04	0.043039
ENSBTAG00000007785	PKD2L2	-1.04	0.004015
ENSBTAG00000011209	SYT11	-1.05	0.022173
ENSBTAG00000007642	ADORA3	-1.05	0.048504
ENSBTAG00000018864	CENPU	-1.06	0.043559
ENSBTAG00000017508	CYSLTR2	-1.06	0.010299
ENSBTAG00000020710	CENPQ	-1.07	0.004250
ENSBTAG00000038340	CLNK	-1.08	0.013924
ENSBTAG00000015177	PRSS23	-1.09	0.005956
ENSBTAG00000012007	SOCS2	-1.09	0.008841
ENSBTAG00000006447	ACSM3	-1.09	0.022324
ENSBTAG00000012907	ODF2L	-1.12	0.001956
ENSBTAG00000011227	TRPC6	-1.13	0.017826
ENSBTAG00000019048	novel gene	-1.14	0.016115
ENSBTAG00000021361	SAMD3	-1.15	0.007383
ENSBTAG00000019670	SUCNR1	-1.15	0.038069
ENSBTAG00000016770	GAPT	-1.15	0.040725
ENSBTAG00000040281	novel gene	-1.17	0.034633
ENSBTAG00000018703	OSTN	-1.17	0.012620
ENSBTAG00000008059	CHRM3	-1.18	0.030701
ENSBTAG00000011990	ALOX15	-1.19	0.045592
ENSBTAG00000042616	U6	-1.20	0.044830
ENSBTAG00000035129	novel gene	-1.22	0.042822
ENSBTAG00000001359	B3GNT7	-1.22	0.024187
ENSBTAG00000019368	IGFBP7	-1.22	0.016306
ENSBTAG00000038141	novel gene	-1.23	0.012135
ENSBTAG00000004917	KLRK1	-1.25	0.012083
ENSBTAG00000047142	LOC784249	-1.26	0.018130
ENSBTAG00000014750	EPB41L4A	-1.26	0.040554
ENSBTAG00000010728	RAB44	-1.27	0.025130
ENSBTAG00000018206	NIPAL2	-1.28	0.001474
ENSBTAG00000018446	GCA	-1.28	0.001474
ENSBTAG00000020602	IDO1	-1.29	0.003116
ENSBTAG00000012991	PRUNE2	-1.30	0.003896
ENSBTAG00000030426	LOC100848575	-1.33	0.048110
ENSBTAG00000020319	ALOX5	-1.33	0.035004
ENSBTAG00000017769	RIBC1	-1.34	0.034151
ENSBTAG00000016548	TEX12	-1.34	0.040346
ENSBTAG00000013550	PRKCG	-1.35	0.022408
ENSBTAG00000038981	LOC518134	-1.35	0.005734
ENSBTAG00000045985	novel gene	-1.35	0.019807
ENSBTAG00000004221	ESM1	-1.46	0.005851
ENSBTAG00000015483	CCR8	-1.47	0.000072
ENSBTAG00000043985	DACH1	-1.47	0.034795
ENSBTAG00000007109	ASB2	-1.47	0.004819

ENSBTAG00000004263	ATP6V0A4	-1.48	0.000055
ENSBTAG00000006643	C6H4orf19	-1.48	0.018149
ENSBTAG00000010057	GZMB	-1.50	0.034924
ENSBTAG00000001125	ADAMDEC1	-1.51	0.049494
ENSBTAG00000020467	HRH4	-1.52	0.000332
ENSBTAG00000040367	novel gene	-1.54	0.017595
ENSBTAG00000000273	IL5RA	-1.56	0.020815
ENSBTAG00000009733	FBP1	-1.56	0.004919
ENSBTAG00000047426	LYPD6	-1.59	0.029108
ENSBTAG00000038080	LOC508858	-1.61	0.010271
ENSBTAG00000025942	HPGD	-1.62	0.019816
ENSBTAG00000031265	SVOPL	-1.63	0.006535
ENSBTAG00000018571	IL1RL1	-1.66	0.001304
ENSBTAG00000013055	novel gene	-1.71	0.002045
ENSBTAG00000046693	novel gene	-1.74	0.003661
ENSBTAG00000017448	EFEMP1	-1.75	0.011716
ENSBTAG00000010828	LOC617313	-1.77	0.002929
ENSBTAG00000038159	novel gene	-1.81	0.009257
ENSBTAG00000015118	PNPLA1	-1.82	0.048266
ENSBTAG00000014831	PPP1R3C	-1.86	0.048110
ENSBTAG00000046668	ANG2	-1.93	0.028869
ENSBTAG00000009252	KLRA1	-1.95	0.025779
ENSBTAG00000046389	KLRD1	-2.02	0.000984
ENSBTAG00000002092	PI16	-2.03	0.018130
ENSBTAG00000014835	SPARC	-2.28	0.045637
ENSBTAG00000020990	P2RY14	-2.30	0.000052
ENSBTAG00000003440	novel gene	-2.35	0.005161
ENSBTAG00000038843	NKG2C	-2.76	0.004285
ENSBTAG00000037539	LOC534578	-4.98	0.021329

Table S2. Differentially expressed genes in the exposed subject when compared with the control group. Genes with an FDR<0,05 and a Log2 fold change (Log2FC) <-1 or >1 were considered as differentially expressed.

Sequence ID	Gene symbol	LogFC	FDR
ENSBTAG00000034449	novel gene	2.87	0.015412
ENSBTAG00000043250	7SK	2.76	0.025306
ENSBTAG00000021807	TAC3	2.52	0.039820
ENSBTAG00000000820	GNG11	2.48	0.020643
ENSBTAG00000004526	TPT1	2.12	0.000000
ENSBTAG00000031849	TMEM119	2.09	0.007448
ENSBTAG00000011299	novel gene	2.09	0.000000
ENSBTAG00000035654	novel gene	1.96	0.009059
ENSBTAG00000047836	novel gene	1.88	0.000006
ENSBTAG00000020368	novel gene	1.87	0.013113

ENSBTAG00000046232	novel gene	1.83	0.009081
ENSBTAG00000032702	novel gene	1.80	0.000000
ENSBTAG00000009737	LTC4S	1.78	0.040494
ENSBTAG00000046100	novel gene	1.76	0.000000
ENSBTAG00000031795	novel gene	1.69	0.012842
ENSBTAG00000004472	DYNLT1	1.65	0.041565
ENSBTAG00000005142	RPL37	1.61	0.032244
ENSBTAG00000040210	novel gene	1.61	0.000253
ENSBTAG00000038913	novel gene	1.60	0.015667
ENSBTAG00000013358	RPS23	1.59	0.015028
ENSBTAG00000022275	novel gene	1.57	0.004814
ENSBTAG00000024950	ACE	1.57	0.017186
ENSBTAG00000012344	RPL26	1.56	0.000920
ENSBTAG00000045673	LOC613345	1.52	0.006652
ENSBTAG00000012219	CSPG4	1.52	0.039730
ENSBTAG00000043582	novel gene	1.46	0.045940
ENSBTAG00000047926	OVCA2	1.45	0.025667
ENSBTAG00000015405	DCHS1	1.43	0.007710
ENSBTAG00000033887	RPL36A-HNRNPH2	1.40	0.006643
ENSBTAG00000037945	CASKIN1	1.37	0.018330
ENSBTAG00000003086	RPLP2	1.36	0.021136
ENSBTAG00000025595	novel gene	1.33	0.013113
ENSBTAG00000026489	TNFRSF13C	1.26	0.000217
ENSBTAG00000046486	novel gene	1.23	0.000253
ENSBTAG00000039470	novel gene	1.22	0.047849
ENSBTAG00000046140	NPDC1	1.21	0.005357
ENSBTAG00000011680	PAPLN	1.19	0.013539
ENSBTAG00000043971	NOTCH3	1.17	0.040494
ENSBTAG00000014358	EVA1B	1.17	0.007790
ENSBTAG00000006368	N4BP3	1.11	0.011447
ENSBTAG00000047508	ZNF580	1.10	0.009059
ENSBTAG00000003675	ADGRL1	1.09	0.003241
ENSBTAG00000021338	OAF	1.08	0.041943
ENSBTAG00000005934	TTYH3	1.08	0.000463
ENSBTAG00000038055	KLF16	1.08	0.000217
ENSBTAG00000015551	RPL15P	1.08	0.015028
ENSBTAG00000019844	ZNF467	1.07	0.000967
ENSBTAG00000026505	novel gene	1.04	0.003884
ENSBTAG00000019097	CNTNAP1	1.03	0.041565
ENSBTAG00000019718	RPS15	1.02	0.003778
ENSBTAG00000002550	novel gene	1.01	0.012842
ENSBTAG00000018554	novel gene	1.01	0.007737
ENSBTAG00000021755	novel gene	1.01	0.002418
ENSBTAG00000019912	CNTD1	-1.00	0.004956
ENSBTAG00000019612	RNASE4	-1.00	0.002928
ENSBTAG00000009156	CAMK2N1	-1.04	0.045243

ENSBTAG00000016415	LTA4H	-1.06	0.000615
ENSBTAG00000030434	FUCA1	-1.07	0.015667
ENSBTAG00000009863	BHLHE40	-1.08	0.000001
ENSBTAG00000021361	SAMD3	-1.14	0.018330
ENSBTAG00000020467	HRH4	-1.18	0.018330
ENSBTAG00000007490	SULF2	-1.19	0.007106
ENSBTAG00000046323	novel gene	-1.21	0.026782
ENSBTAG00000038340	CLNK	-1.28	0.008956
ENSBTAG00000018446	GCA	-1.29	0.003778
ENSBTAG00000020602	IDO1	-1.31	0.005843
ENSBTAG00000038141	novel gene	-1.35	0.012842
ENSBTAG00000019368	IGFBP7	-1.38	0.018655
ENSBTAG00000003297	OASL	-1.39	0.030394
ENSBTAG00000004221	ESM1	-1.43	0.018655
ENSBTAG00000009733	FBP1	-1.43	0.025667
ENSBTAG00000018499	ANXA8L1	-1.46	0.006894
ENSBTAG00000010728	RAB44	-1.46	0.025306
ENSBTAG00000015177	PRSS23	-1.48	0.000296
ENSBTAG00000015483	CCR8	-1.49	0.000161
ENSBTAG00000018571	IL1RL1	-1.58	0.007106
ENSBTAG00000004263	ATP6V0A4	-1.60	0.000018
ENSBTAG00000010828	LOC617313	-1.77	0.006833
ENSBTAG00000046158	CFB	-1.78	0.027565
ENSBTAG00000046693	novel gene	-1.78	0.008086
ENSBTAG00000013055	novel gene	-1.85	0.001996
ENSBTAG00000031265	SVOPL	-1.85	0.005357
ENSBTAG00000047632	IGHE	-1.90	0.000040
ENSBTAG00000020990	P2RY14	-1.91	0.003211
ENSBTAG00000010057	GZMB	-1.94	0.013539
ENSBTAG00000007109	ASB2	-1.97	0.000198
ENSBTAG00000038159	novel gene	-1.97	0.011841
ENSBTAG00000047449	LOC100300483	-1.98	0.000253
ENSBTAG00000001125	ADAMDEC1	-2.00	0.018330
ENSBTAG00000047816	novel gene	-2.10	0.000217
ENSBTAG00000038080	LOC508858	-2.11	0.001240
ENSBTAG00000032051	LOC100300896	-2.14	0.009767
ENSBTAG00000001292	LTF	-2.34	0.002418
ENSBTAG00000002092	PI16	-2.36	0.014950

Table S3. **Differentially expressed genes in the positive subject when compared with the exposed group.** Genes with an FDR<0,05 and a Log2 fold change (Log2FC) <-1 or >1 were considered as differentially expressed.

Sequence ID	Gene symbol	LogFC	FDR
ENSBTAG00000047632	IGHE	1.71	0.006138
ENSBTAG00000039555	LOC101902937	-2.69	0.036802

Table S4. List of 123 transcripts identified by Combinatorial Discriminant Analysis (CDA) that discriminates between cattle infected and non-infected by *Mycobacterium avium ssp. Paratuberculosis.*

**Probe Id**
ENSBTAG00000000030
ENSBTAG00000000031
ENSBTAG00000000140
ENSBTAG00000000147
ENSBTAG00000000241
ENSBTAG00000000256
ENSBTAG00000000273
ENSBTAG00000000289
ENSBTAG00000000332
ENSBTAG00000000409
ENSBTAG00000000013
ENSBTAG00000000014
ENSBTAG00000000016
ENSBTAG00000000019
ENSBTAG00000000021
ENSBTAG00000000023
ENSBTAG00000000024
ENSBTAG00000000025
ENSBTAG00000000032
ENSBTAG00000000033
ENSBTAG00000000037
ENSBTAG00000000049
ENSBTAG00000000050
ENSBTAG00000000054
ENSBTAG00000000056
ENSBTAG00000000057
ENSBTAG00000000065
ENSBTAG00000000067
ENSBTAG00000000072
ENSBTAG00000000073
ENSBTAG00000000077

ENSBTAG00000000084
ENSBTAG00000000087
ENSBTAG00000000088
ENSBTAG00000000090
ENSBTAG00000000094
ENSBTAG00000000098
ENSBTAG00000000105
ENSBTAG00000000108
ENSBTAG00000000115
ENSBTAG00000000130
ENSBTAG00000000131
ENSBTAG00000000132
ENSBTAG00000000134
ENSBTAG00000000146
ENSBTAG00000000154
ENSBTAG00000000156
ENSBTAG00000000158
ENSBTAG00000000160
ENSBTAG00000000161
ENSBTAG00000000162
ENSBTAG00000000163
ENSBTAG00000000175
ENSBTAG00000000179
ENSBTAG00000000181
ENSBTAG00000000184
ENSBTAG00000000186
ENSBTAG00000000191
ENSBTAG00000000199
ENSBTAG00000000202
ENSBTAG00000000203
ENSBTAG00000000204
ENSBTAG00000000205
ENSBTAG00000000211
ENSBTAG00000000212
ENSBTAG00000000215
ENSBTAG00000000231
ENSBTAG00000000240
ENSBTAG00000000242
ENSBTAG00000000245
ENSBTAG00000000246
ENSBTAG00000000251
ENSBTAG00000000253
ENSBTAG00000000260
ENSBTAG00000000266

ENSBTAG00000000267
ENSBTAG00000000269
ENSBTAG00000000271
ENSBTAG00000000274
ENSBTAG00000000275
ENSBTAG00000000277
ENSBTAG00000000279
ENSBTAG00000000281
ENSBTAG00000000283
ENSBTAG00000000285
ENSBTAG00000000286
ENSBTAG00000000287
ENSBTAG00000000291
ENSBTAG00000000292
ENSBTAG00000000296
ENSBTAG00000000297
ENSBTAG00000000301
ENSBTAG00000000306
ENSBTAG00000000312
ENSBTAG00000000313
ENSBTAG00000000315
ENSBTAG00000000317
ENSBTAG00000000320
ENSBTAG00000000341
ENSBTAG00000000342
ENSBTAG00000000345
ENSBTAG00000000355
ENSBTAG00000000356
ENSBTAG00000000357
ENSBTAG00000000359
ENSBTAG00000000362
ENSBTAG00000000365
ENSBTAG00000000369
ENSBTAG00000000371
ENSBTAG00000000375
ENSBTAG00000000376
ENSBTAG00000000377
ENSBTAG00000000379
ENSBTAG00000000380
ENSBTAG00000000381
ENSBTAG00000000386
ENSBTAG00000000387
ENSBTAG00000000390
ENSBTAG00000000393

ENSBTAG00000000399
ENSBTAG00000000404
ENSBTAG00000000405
ENSBTAG00000000406
